# Incubation Temperature Effects on Hatchling Performance in the Loggerhead Sea Turtle (*Caretta caretta*)

**DOI:** 10.1371/journal.pone.0114880

**Published:** 2014-12-17

**Authors:** Leah R. Fisher, Matthew H. Godfrey, David W. Owens

**Affiliations:** 1 University of Charleston South Carolina at the College of Charleston, Charleston, South Carolina, United States of America; 2 North Carolina Wildlife Resources Commission, Beaufort, North Carolina, United States of America; 3 Duke University Marine Lab, Nicholas School of the Environment, Beaufort, North Carolina, United States of America; Institute of Marine Research, Norway

## Abstract

Incubation temperature has significant developmental effects on oviparous animals, including affecting sexual differentiation for several species. Incubation temperature also affects traits that can influence survival, a theory that is verified in this study for the Northwest Atlantic loggerhead sea turtle (*Caretta caretta*). We conducted controlled laboratory incubations and experiments to test for an effect of incubation temperature on performance of loggerhead hatchlings. Sixty-eight hatchlings were tested in 2011, and 31 in 2012, produced from eggs incubated at 11 different constant temperatures ranging from 27°C to 33°C. Following their emergence from the eggs, we tested righting response, crawling speed, and conducted a 24-hour long swim test. The results support previous studies on sea turtle hatchlings, with an effect of incubation temperature seen on survivorship, righting response time, crawling speed, change in crawl speed, and overall swim activity, and with hatchlings incubated at 27°C showing decreased locomotor abilities. No hatchlings survived to be tested in both years when incubated at 32°C and above. Differences in survivorship of hatchlings incubated at high temperatures are important in light of projected higher sand temperatures due to climate change, and could indicate increased mortality from incubation temperature effects.

## Introduction

Temperature plays a critical role in animal development. In particular, incubation temperature has significant developmental effects on oviparous animals, including on the direction of sexual differentiation for several species [10]. Temperature-dependent sex determination (TSD) is found in seven orders of fish [9], and many reptiles: all crocodilians, tuataras, many turtles, and some lizards (reviewed in [54]). All sea turtle species exhibit TSD, with higher temperatures producing more to all females, and cooler temperatures producing more to all males (reviewed in [55]). TSD is a type of environmental sex determination (ESD), and contrasts with the more common genetic sex determination (GSD).

For reptiles with TSD, climate change impacts, particularly rising temperatures around the world, dictate a need for an improved understanding of how even subtle temperature changes in the nest environment will influence the survivorship or fitness of both individuals and species in coming decades. However, both survivorship and fitness of sea turtles in terms of lifetime fecundity are nearly impossible to test directly, due to their slow growth and late maturity [21]. Thus, we use phenotypic measures such as crawling speed, self-righting time, and swimming performance as a proxy for hatchling fitness, as has become standard practice in studies on sea turtle hatchlings [5], [6], [7], [29], [40], [47], [52], [58].

### Incubation Temperature Effects on Performance

Incubation temperature influences several factors in sea turtle egg incubation besides hatchling sex, including development time, size, mass, and amount of yolk content converted to hatchling tissue [4], [14], [48]. Incubation temperature can also affect locomotor performance for hatchling sea turtles [5], [6], [7], [29], [39], [47], [58]. It is generally assumed that an increase in locomotor activity, for example crawling faster or swimming longer, is advantageous because it may allow hatchlings to better avoid predators [30]. In turn, this increased performance could affect lifetime fitness. Studies on freshwater turtle species have seen a lasting effect of incubation temperature on various measures even after six months to a year [1], [15], [46].

For freshwater turtles, it has been reported that hatchlings show decreased performance ability at the upper and lower extremes of incubation temperature [4], [15], [40]. For loggerhead (*Caretta caretta*), green (*Chelonia mydas*), and olive ridley (*Lepidochelys olivacea*) sea turtles, several studies [2], [3], [4], [6], [21], [29], [39], [47], [58] have found that hatchlings produced at warmer temperatures may be smaller in carapace dimensions, and that incubation temperature affects hatchling locomotor performance and possibly even growth rates [17]. However, these studies did not test the upper limit of incubation temperature, and the fitness consequences of differences in hatchling size and locomotor performance remain untested [26].

More specifically for green sea turtles, hatchlings incubated at 26°C were seen to have lower flipper stroke rate frequency and force output than hatchlings incubated at 28°C and 30°C [4], [7]. This suggests that male hatchlings incubated at the cooler extreme of incubation temperature have decreased locomotor ability compared to hatchlings incubated at mid-range temperatures. For loggerhead hatchlings in Australia and New Caledonia born on a natural beach, two studies have reported decreased locomotor performance when incubated at the warmer extreme of incubation temperature [47], [58]. These results suggest that sea turtle hatchlings show optimal performance when incubated at mid-range temperatures. However, there has not been a controlled incubation experiment conducted on loggerhead hatchlings measuring potential fitness consequences from physical differences based on incubation temperature. A study designed to test physical and behavioral differences of loggerhead hatchlings incubated at a wide range of temperatures is needed.

### Climate Change Connection

The fate of the Atlantic loggerhead sea turtle is uncertain. Anthropogenic threats combined with the impacts of rapid global climate change could have a major impact on the population of this species [16]. Sea turtles are long-lived, slow growing, and late maturing animals, and in the past they have been able to adapt to a changing climate, but with a significantly slower rate of climate change than is projected for the next 100 years [21]. There are concerns that rapid climate change will have negative impacts on the populations of this species [26]. Research focusing on the potential effects of climate change is important and necessary to better understand, manage, and predict the population dynamics of this long-lived reptile into the future.

Rising sand temperatures on beaches are one way climate change may already be impacting sea turtle populations around the world. Temperatures at many nesting beaches worldwide have already been seen to be warming, for example in the Caribbean [18], South Atlantic [25], and Western Pacific [8]. Due to TSD, there is a concern for all sea turtle species that the female to male sex ratio in the population is increasing and may approach 100% female, and that sea turtles will be unable to adapt at the rate needed to combat rapid climate change [26]. This concern is exacerbated by the knowledge that sea turtles are imprinted to their natal beach region, and thus may not have the ability to adapt to climate shifts as rapidly as can other species that do not exhibit natal homing [22]. Furthermore, with extreme incubation temperatures resulting in a decrease in performance of hatchlings, as seen for both freshwater and sea turtles, hot beach temperatures could decrease overall hatchling survival [4], [5], [6], [7], [15], [29], [37], [38], [39], [40], [47], [58].

### Controlled Incubation Experiment Needed

Our main goal in this study was to test for performance and survivorship differences between loggerhead hatchlings incubated at different controlled temperatures. A standardized laboratory experiment that incubates loggerhead eggs across the viable incubation temperature range can investigate potential incubation temperature effects on hatchling fitness. Examples of performance that indicate survival ability of hatchlings include the righting response [12], [53], crawling speed [14], swimming stroke rate frequency, and overall swimming activity [49], [59]. The righting response refers to the ability of a hatchling to turn itself over after being placed upside down on its carapace [12]. The other locomotor functions of crawling and swimming are considered variables indicating hatchlings' ability to reach their offshore nursery grounds by escaping the predator-laden beach and near-shore predator-rich waters [20].

## Materials and Methods

### Egg Collection and Incubation Protocol

Laboratory experiments were conducted to test for an effect of incubation temperature on performance of 68 loggerhead hatchlings in 2011 and 31 in 2012. The methods were held constant between the two years, and any differences that arose during the course of the experiments will be specifically described. This study was carried out with the approval of the Institutional Animal Care and Use Committee (College of Charleston protocol #2011-005; North Carolina State University protocol #11-078-O), and authorization to collect eggs in the field was permitted by the South Carolina Department of Natural Resources.

The hatchlings tested came from three loggerhead clutches collected the morning after they were laid on North Island, South Carolina (Yawkey Wildlife Center) on 20 June 2011 (Clutch A, 89 eggs), 22 June 2011 (Clutch B, 109 eggs), and 21 May 2012 (Clutch C, 112 eggs). On the beach, volunteers monitoring nesting for the South Carolina Department of Natural Resources (SC-DNR) collected the eggs after daybreak, after they had been laid the previous night. Eggs were placed into buckets and insulated with sand from the nest site for transport on foot to a boat, and then transferred to a car. Each clutch was transported in a single bucket. The clutches were driven to the Center for Marine Sciences and Technology (CMAST) in Morehead City, North Carolina, where they were incubated. Total time between laying and placement in incubators was about 18 hours, assuming that nests were laid around midnight [43].

The eggs from each clutch were distributed randomly between five incubators set to temperatures ranging from 27°C–32.5°C. Because of the slight variation in temperature among the shelves within the incubators, we maintained an approximately equal number of eggs from each clutch on each shelf of the incubators. For each clutch, there were five to eight eggs per shelf, with a total of ten to sixteen eggs per shelf. Each egg was incubated individually in a labeled white plastic cup, sitting on a synthetic sponge, surrounded by moist vermiculite (for details, [44]). Each shelf had an Hg thermometer, calibrated against a standard Hg thermometer, immersed in glycerin to facilitate real-time data recording, and the temperature of each shelf was recorded daily. At days 15–17 and 38–40 of incubation, ∼65 mL of distilled water were added to the vermiculite around each egg.

In 2011, one incubator experienced high temperatures during the middle of incubation, and data are excluded from this incubator. Excluding this incubator, no shelf temperature varied more than 0.5°C during incubation. The average temperature of the shelves in the four incubators varied, and eggs experiencing similar temperatures were grouped together. Final experimental temperature groups were 27, 28, 28.5, 29, 29.5, 30, 31, 32, and 32.5°C. In 2012, temperature variations per shelf never exceeded 0.5°C. The experimental temperature groups in 2012 were 26.5, 27.5, 29, 29.5, 30, 30.5, 32.5, and 33°C.

### Hatching Protocol

A turtle was considered “hatched” when it had its head and at least one flipper out of the egg [19]. After 24–36 hours, the hatchling was removed from its egg, measured and weighed, labeled, and then placed in an individual holding container in a separate incubator.

Curved carapace length and curved carapace width were recorded using a measuring tape to the nearest 0.1 cm, and weight was recorded to 0.01 g. A unique ID was painted on the carapace scutes with non-toxic white paint. The holding containers were plastic cups surrounded in tinfoil to keep the hatchlings in the dark, and the holding incubator was set to 29.5°C. This simulated the natural warm and dark environment in a nest that a hatchling experiences before emerging (Wyneken pers. comm.). Hatchlings remained in the containers for a minimum of 72 hours and a maximum of 96 hours to simulate the actual course of hatching and emergence from a nest [19], [43]. This three to four day period corresponds to the time a hatchling spends internalizing residual yolk and straightening its carapace [19]. After this time period, hatchlings were transported in their individual holding containers by car 33.8 km to the Marine Aquaculture Research Center (MARC) facility in Marshallburg, North Carolina. Experiments took place from 7 August–6 September, 2011, and from 15 June–3 August, 2012.

### Experimental Protocol

Experiments started two hours after hatchlings were removed from the holding incubator. All tests were conducted in one room in the MARC facility that was climate controlled, and air temperature in the room was maintained between 24°C–26°C to approximate nighttime air temperatures. Hatchlings were tested one by one, and up to ten hatchlings could be tested per trial. The order of hatchlings tested in a trial was random. There were 10 experimental trials in 2011, and 8 trials in 2012, grouped by hatching order. The trial groupings roughly corresponded to incubation temperature groupings of hatchlings, due to the fact that incubation temperature determines incubation duration [14]. Before the tests, non-toxic whiteout dots were placed on the ends of the two front flippers to facilitate the video analysis of the swim test, specifically for easier differentiation of swim behavior. For each hatchling, three tests were conducted in this order: righting response, crawl, and swim test.

### Righting Response and Crawl Test

Hatchlings were monitored for righting response and the crawl test on a 0.3 m×2.44 m runway. The runway was set at an angle of 25° to simulate the inclination of a natural beach, and lined with sand-colored carpet. The first test was the righting response. A hatchling was placed upside down on its carapace at one end of the runway. As soon as the hatchling was set down, we used a stopwatch to record how many seconds it took the hatchling to flip itself over. This was repeated four times for each hatchling. If after two minutes the hatchling could not turn itself over, this was noted and the hatchling was manually turned over.

Immediately after the hatchling righted itself for the fourth time, the crawl test commenced. The hatchling would crawl from the top of the runway to the bottom, where an attached night-light served as the only light source for hatchling orientation. On a natural beach, hatchlings crawl towards the brightest light source, which is over the ocean horizon when there are no artificial lights on a beach [56]. The time each hatchling took to move from one marked end of the runway to the other was recorded in seconds. This process was repeated three times with no breaks for a total distance crawled of 7.32 meters, and mean speed calculated for each run and for all three runs together (modified from [14]). The hatchling then immediately started the swim test, simulating what happens naturally post-emergence as hatchlings crawl down the beach to reach the ocean.

### Swim Test

The methodology for the swim test takes advantage of the hatchling swimming frenzy that occurs continuously for the first approximately 24 hours in the ocean (methods derived from [49]). Each hatchling was fitted with a black lycra harness closed with Velcro that did not impede motion. This harness was attached to the center of a monofilament line tied across the center of a 54 cm diameter bucket. This served as a tether, allowing the hatchling to swim in any direction without touching the bottom or sides of the bucket, thereby simulating an open-water oceanic environment [49]. Each hatchling swam in its own circular 64.35 L bucket that contained filtered seawater maintained at a constant ∼26°C with a flow-through water system.

As soon as the first hatchling was hooked into its harness, two Axis P1346 video surveillance cameras mounted 2.1 meters above the buckets started recording at 30 frames per second. Each camera recorded five buckets at a time, allowing up to ten hatchlings to be tested simultaneously per trial. Recordings lasted for 24 hours after the last hatchling started swimming. Night-lights mounted on the wall opposite the buckets, at 15° and 30° above the hatchlings, served as an orientation light cue for the hatchlings. The only other light sources were three high-powered infrared lamps spread throughout the room to enhance the video recordings. Infrared lamps were used because loggerheads do not respond to infrared wavelengths [35]. The experiment started with 12 hours of darkness to simulate natural events, that hatchlings typically enter the ocean at night after emerging from the nest. After 12 hours, the overhead fluorescent lights in the room turned on for 12 hours of “daytime”.

### Video Analysis

In 2011, we conducted video analysis of the swim test by watching all of the recordings at 4× speed. We found that when 10 minutes of every hour of video were analyzed, compared to the entire hour, the results were identical. Thus, when analyzing the swim data from 2012, we analyzed the first ten minutes of every hour of video.

Following the preliminary viewing of the videos in 2011, we developed a categorical index, or ethogram, to describe 98% of the swimming behaviors observed for the hatchlings: PowerStroke (PS), Rear-Flipper Kick (RFK), Mixed Rest (MR), and Rest (R). PS describes the most powerful swimming behavior, when turtles use both of their front flippers to propel through the water, while RFK refers to when they are only moving by using their back flippers (described in [49]). MR was a mix of PS, RFK, breathing, and/or R behaviors, with all of these behaviors lasting less than 30 seconds. Rest (R) was simply when hatchlings were not moving any flippers. These categories and the times of their occurrence were recorded as the videos were watched. Any change in swimming behavior that lasted longer than 30 seconds was recorded. Breaths, sometimes described as dog paddling, that lasted longer than 15 seconds were also recorded, along with any interference to the hatchlings (for example, having to re-attach a harness). Overall swimming activity was quantified as [%PS]+[%RFK] over 24 hours, because these were the two swim behaviors when hatchlings exhibited oriented swimming. We also noted when hatchlings made their first major switch from PS to another form of swimming, either RFK, MR, or R, which was defined as equal to or greater than 30 minutes of one hour, a sign of possible tiring during the frenzy.

The analysis of swimming stroke rate frequency was conducted over the first 4 hours of swimming, because this was the time period during which all hatchlings were PowerStroking. This analysis was limited to hatchlings tested in 2011. Flipper stroke rate frequency was calculated by counting strokes for one minute every half hour, resulting in a nine-point time series for each turtle. A proxy for distance was calculated using total percent time PS for each hatchling (%PS×stroke rate frequency), allowing generation of a “Distance” Index.

### Statistical Analysis

Analyses were performed using “R” (R: A Language and Environment for Statistical Computing, R Core Team, 2012). Percent hatched and percent survivorship were converted to binary data and were analyzed using a logistic regression, with incubation temperature as a factor. Performance data from 2011 and 2012 were analyzed separately due to an apparent temporal and/or clutch difference in performance between the two years, and results are also presented separately.

Two-way analyses of variance (ANOVA) were performed separately on the righting response, crawl, and swim data, using both incubation temperature and clutch as factors. In cases where residuals and/or variances were not normally distributed, log-transformations were performed before running the ANOVA. If ANOVA was significant, post-hoc pair-wise comparisons of additional ANOVAs were performed across temperature groups. An analysis of covariance (ANCOVA) was used to analyze potential interactions or relationships between incubation temperature and swimming stroke rate frequency, as well as body mass (2011 only). Change in stroke rate frequency over time was analyzed with multiple regression (2011 only). Finally, we assembled a composite rank value for each hatchling in 2011 by summing the ranks of all statistically significant performance variables. Low ranks were assigned to decreased locomotor abilities; for example, the hatchling that crawled the slowest would have a rank of 1, with ranks then increasing with crawl speed. Several curves were fit to the data, and the best fit was assigned based on a corrected Akaike Information Criterion (AIC_c_) value comparison and Akaike weight calculation [31].

Due to the high number of tests performed on each hatchling, we looked for correlations using pair-wise comparisons of performance variables, specifically between the three groups of tests performed (righting response, crawl, and swim test). There were no statistically significant correlations present, and the highest R^2^ value seen was 0.22. Thus we treated the performance tests as independent of each other, and statistical significance for righting response and crawl was set to p≤0.05. However, for the swim test, we performed a Sidak-Bonferroni Correction to account for the six variables tested on the swim data (overall swim activity, %PS, %RFK, %MR, %R, and switch from PS). The result of this correction was that statistical significance was set to p≤0.0085.

## Results

### Percent Hatched and Survivorship

**Figure 1:**
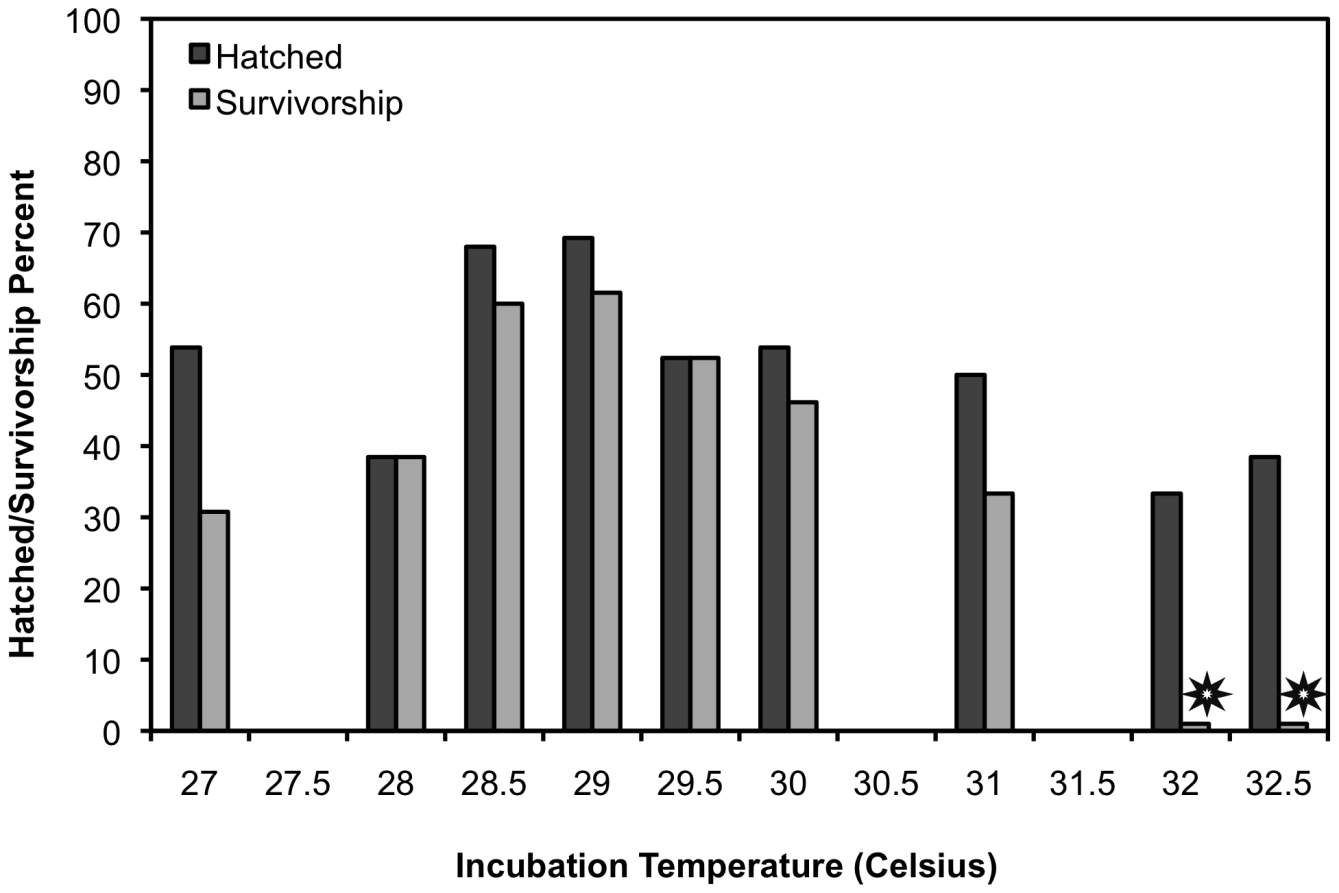
Overall percent of hatched eggs (darker bars) and percent of hatchlings that survived (lighter bars) for the two clutches in 2011. Percent hatched and percent survivorship were maximized at 29°C, with minimums seen at the extremes of incubation temperature. A significant effect of incubation temperature on survivorship was seen at 32 and 32.5°C, with stars denoting statistical significance. The same overall patterns were seen in 2012, but due to an apparent clutch and/or year effect, data were excluded from this figure.

There was one incubator in 2011 that increased its temperature ∼2°C in the middle of the incubation period; these hatchlings were excluded from all analyses. In 2011, there was a 62% hatch success rate (n = 88/143), and 41% survivorship (n = 59/143). The difference in these values came from hatchlings that hatched but then died before starting a trial during the three-day holding period. Percent hatched in 2011 at different temperatures ranged from 33.3% (n = 4/12) at 32°C, to 69.2% (n = 18/26) at 29°C (Fig. 1). There was no significant difference in hatch rates seen across incubation temperatures (Fig. 1).

Incubation temperature did affect percent survivorship of both clutches in 2011, specifically at 32°C, 32.5°C, where no hatchlings survived to be tested (G = 36.61, p<0.0001, Fig. 1). Of the temperatures where hatchlings from Clutch A and B survived to be tested, percent survivorship ranged from 30.7% (n = 4/13) at 27°C to 61.5% (n = 16/26) at 29°C (Fig. 1). There was no significant difference in percent hatched between Clutch A and Clutch B; however, Clutch A had higher survivorship overall (50%) than Clutch B (34%, F_2,1_ = 4.848, p<0.05). Hatchling body mass ranged from 16.00 g to 22.16 g, and there was no detectable effect of incubation temperature on body mass. Curved carapace length (CCL) ranged from 4.2 cm–4.9 cm. Hatchlings were smallest at 27°C and 31°C, and the largest at 29°C, 29.5°C, and 30°C (F_2,6_ = 2.396, p<0.05).

In 2012 Clutch C showed similar results to 2011, but with an overall lower hatching rate: 33.0% hatch success rate (n = 33/112), and 27.7% survivorship (n = 31/112). Percent hatched ranged from 0% (n = 0/20) at 32.5 and 33°C, to 75% (n = 6/8) at 30°C. As evidenced by the 0% hatching at 32.5 and 33°C, Clutch C also had no hatchlings survive when incubated above 32°C, giving incubation temperature a significant effect on survivorship (G = 19.86, p = 0.01).

### 2011: Incubation Temperature Effect on Performance Variables

**Figure 2:**
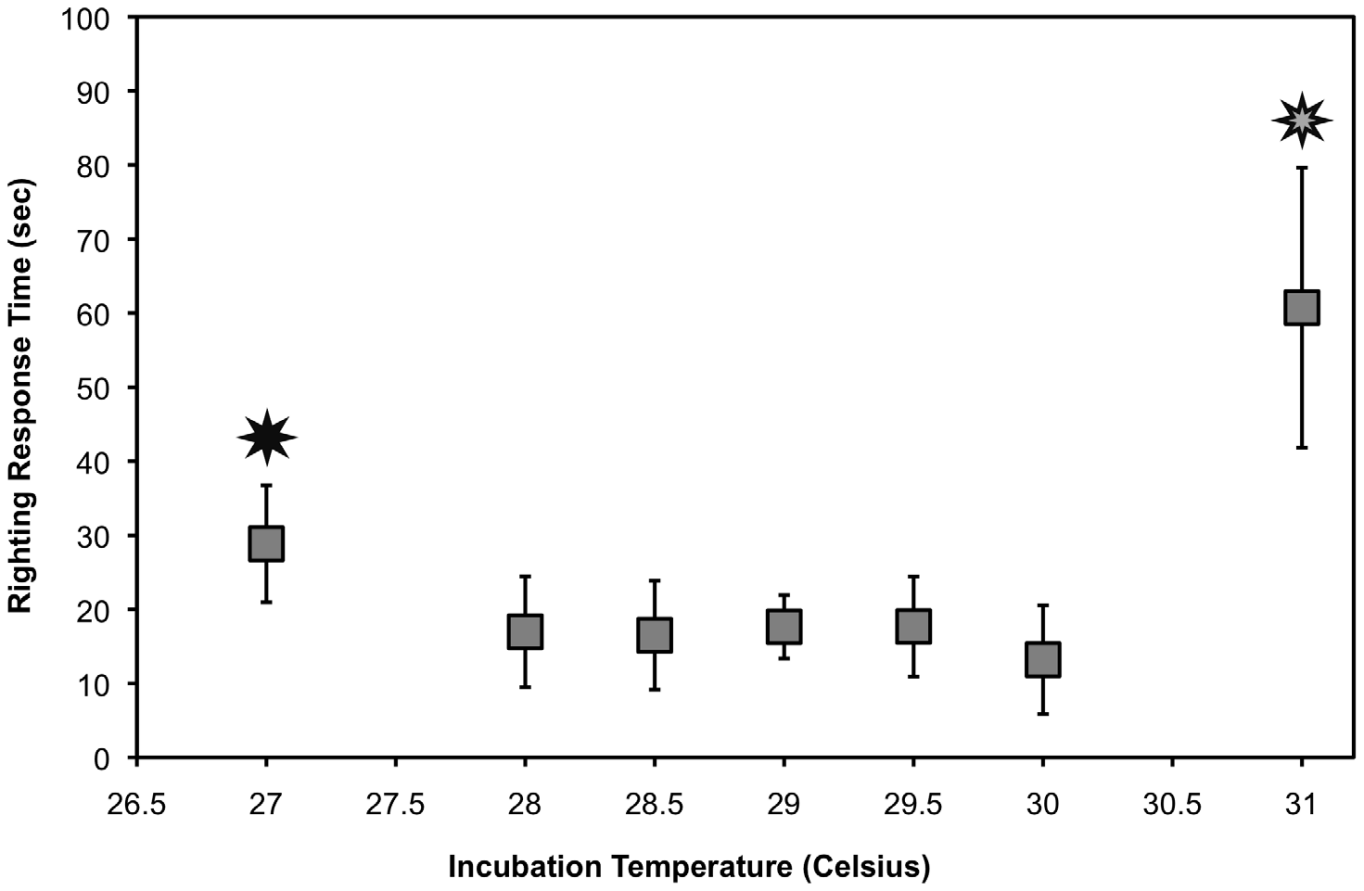
Mean Righting Response time ± S.E.M. in 2011, demonstrating that hatchlings incubated at 27°C and 31°C take significantly longer to right themselves than hatchlings from the middle range of incubation temperatures (F_2,6_ = 2.969, p = 0.01). Hatchlings from 31°C also took significantly longer to right themselves than hatchlings from 27°C. Stars denote statistical significance. [Sample size: 27°C n = 5; 28°C n = 5; 28.5°C n = 12; 29°C n = 15; 29.5 n = 11; 30°C n = 6; 31 n = 4].

**Figure 3:**
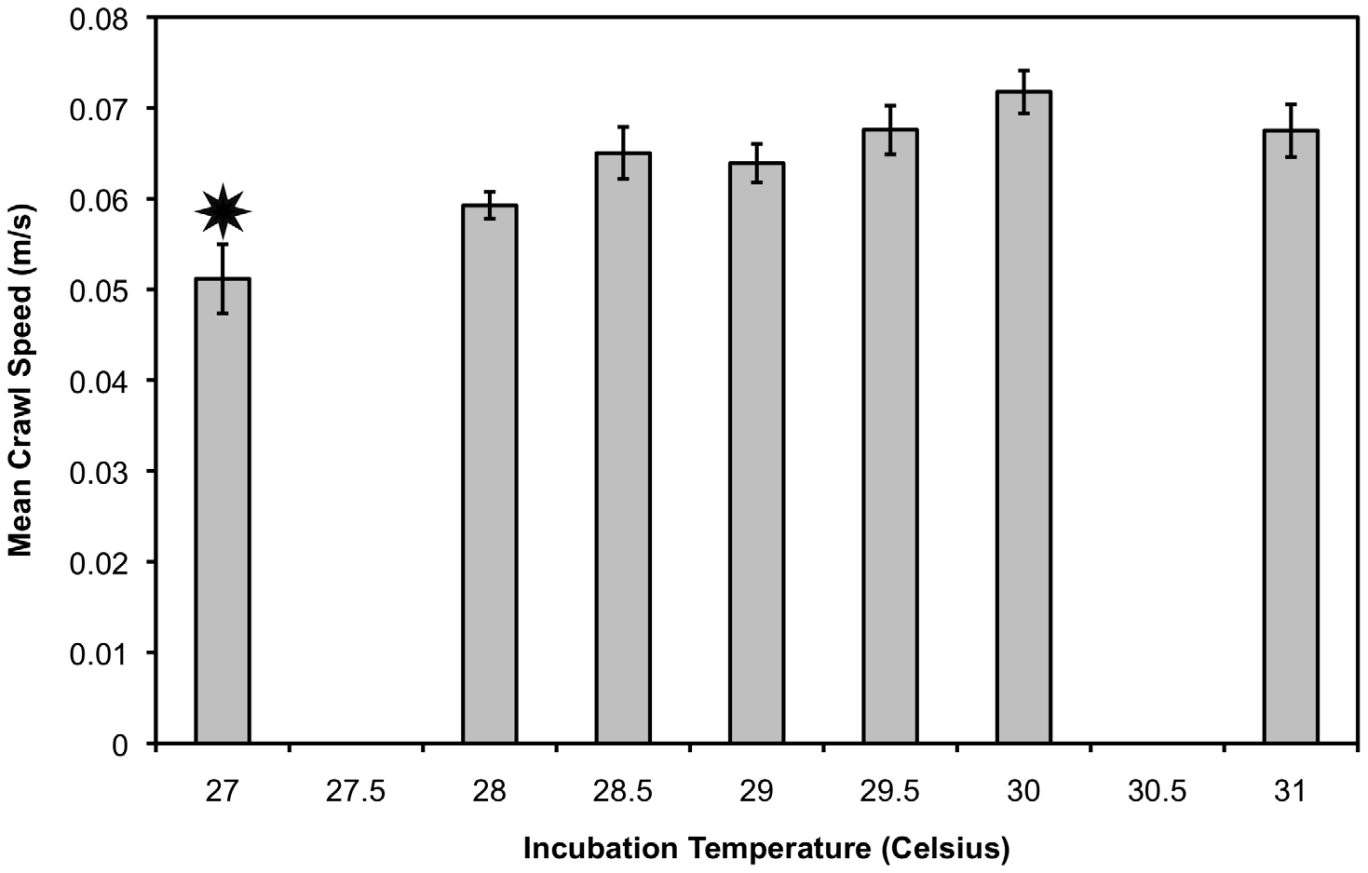
Mean crawl speed for the third crawl (C3) ± S.E.M. in 2011, with hatchlings from 27°C (starred) being significantly slower than all other hatchlings (F_2,6_ = 3.979, p<0.01). This trend is seen for C1 and C2 as well, but the effect of incubation temperature is not detectable statistically. [Sample size: 27°C n = 5; 28°C n = 5; 28.5°C n = 12; 29°C n = 15; 29.5 n = 11; 30°C n = 6; 31 n = 3].

**Figure 4:**
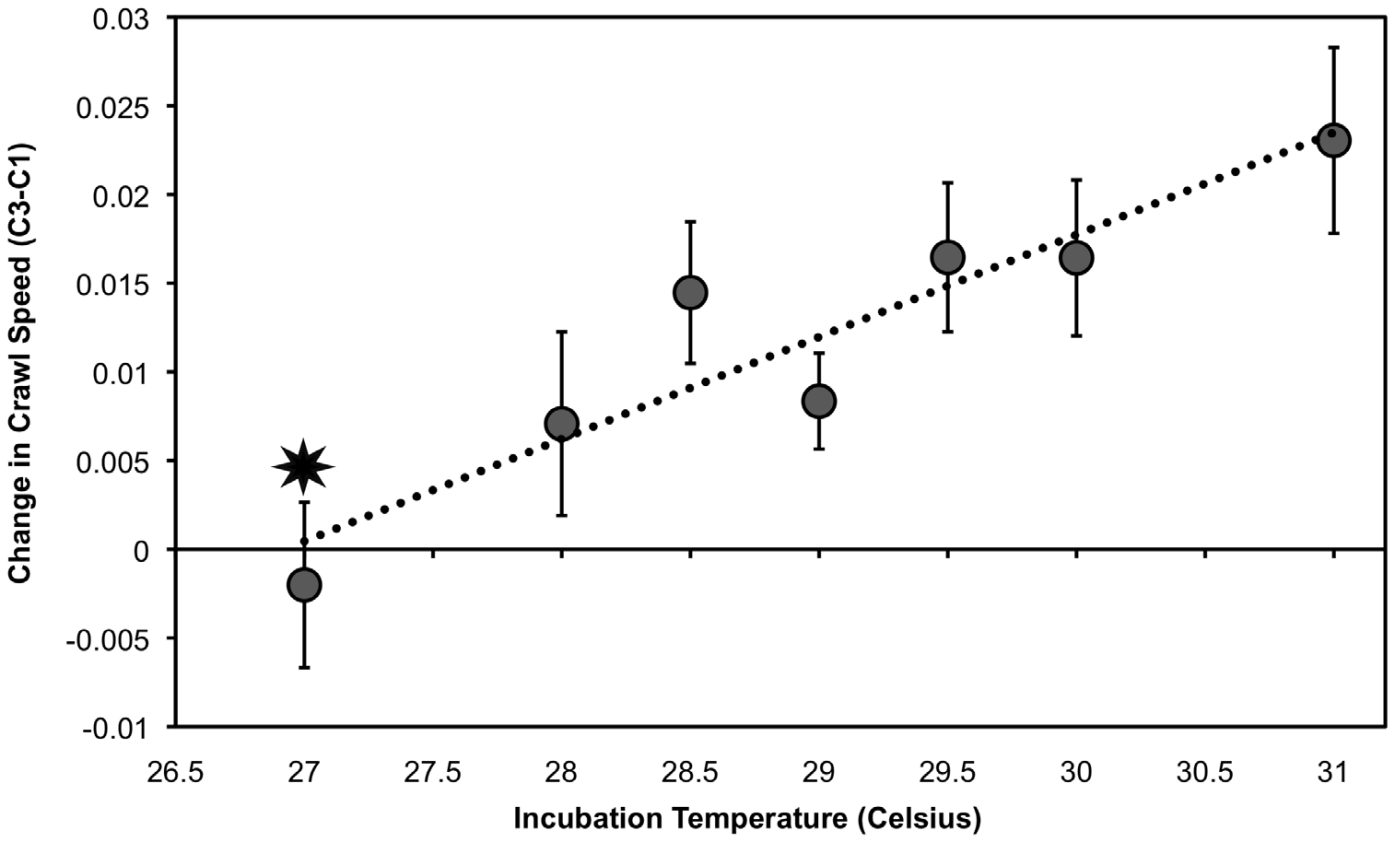
Incubation temperature's effect on mean change in crawl speed (C3-C1) ± S.E.M in 2011. On average, hatchlings from 27°C slowed down while all other hatchlings sped up between crawls (F_2,6_ = 7.010, p = 0.01). Star denotes statistical difference. A linear relationship between these data is presented (R^2^ = 0.87). [Sample size: 27°C n = 5; 28°C n = 5; 28.5°C n = 12; 29°C n = 15; 29.5 n = 11; 30°C n = 6; 31 n = 3].

**Figure 5:**
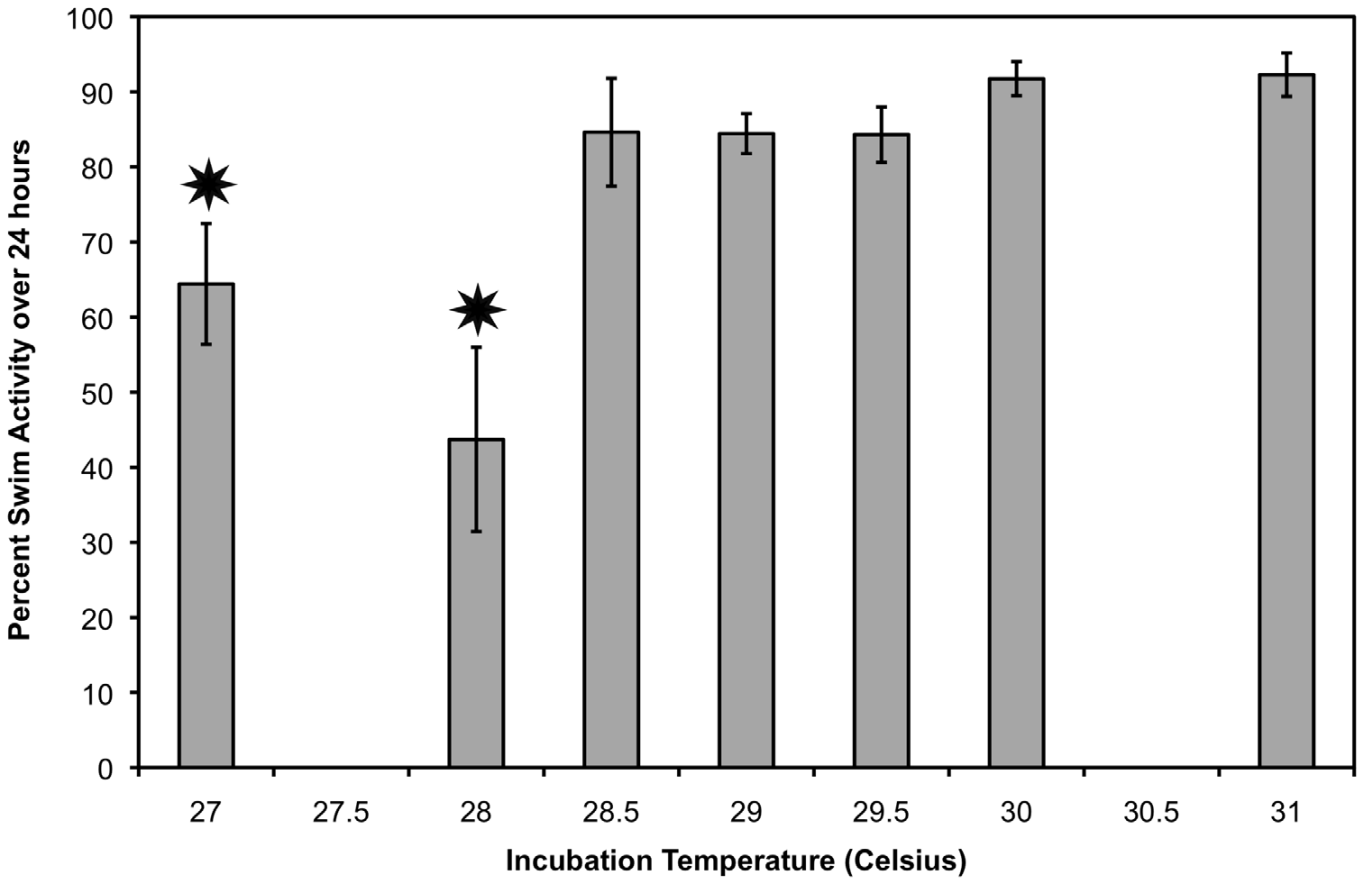
The effect of incubation temperature on overall swimming activity ± S.E.M. in 2011. Hatchlings from 27°C and 28°C (stars) were significantly less active than those from all other incubation temperatures (F_2,6_ = 8.022, p<0.0001). Incubation temperature maintained a significant effect on hatchlings from 27°C when the two hatchlings incubated at 28°C were removed from the analysis. [Sample size: 27°C n = 4; 28°C n = 2; 28.5°C n = 7; 29°C n = 14; 29.5 n = 10; 30°C n = 6; 31 n = 4].

Incubation temperature had a significant effect on mean righting response time (F_2,6_ = 2.969, p = 0.01, Fig. 2), crawl speed for the third crawl (F_2,6_ = 3.979, p<0.01, Fig. 3), change in crawl speed (F_2,6_ = 7.010, p = 0.01, Fig. 4), overall swimming activity (F_2,6_ = 8.022, p<0.0001, Fig. 5), and %MR (F_2,6_ = 4.590, p = 0.001). Hatchlings from 27°C and 31°C took significantly longer to right themselves than hatchlings from the middle incubation temperatures (Fig. 2). Righting response time ranged from 0.65 s to 120 s (maximum time before hatchling was manually turned over). Mean righting response time per individual ranged from 1.50 s to 90.63 s.

Turtle crawl speeds ranged from 0.031 m/s to 0.080 m/s. Incubation temperature had no statistically significant effect on the first or second crawl speeds. During the third crawl (C3) incubation temperature did have a detectable effect, and speeds ranged from 0.040 m/s to 0.080 m/s. Hatchlings produced at 27°C were significantly slower (Fig. 3). Similarly, hatchlings from 27°C seemed to fatigue quicker by slowing down on average between the first and third crawl (C3-C1; Fig. 4). At all other temperature groupings, hatchlings sped up over the three runs, demonstrating a linear increase in change in speed (Fig. 4). Change in speed ranged from slowing down by 0.015 m/s to speeding up by 0.048 m/s.

Overall swimming activity ranged from 31.45% active to 98.31% active. A clear pattern emerged, with most hatchlings spending about 80% to 90% of their time actively swimming; however, hatchlings produced at 27°C and 28°C were significantly less active (Fig. 5). It should be noted that for incubation at 28°C, n = 2, compared to a minimum of n = 4 for all other incubation temperatures. With 28°C excluded from the analysis, incubation temperature maintains a significant effect (F_2,5_ = 6.624, p<0.001).

Incubation temperature had no effect on the other swim test performance variables, including %PS, %RFK, %R, or the first switch from PS to another form of swimming. There was also no effect on stroke rate frequency over the first four hours of PS, nor did incubation temperature affect the “Distance” Index based on stroke rate frequency and %PS. All of the performance results are summarized in Table 1.

**Table 1:** Summary of all 2011 results for Clutch A and B.

	TEMPERATURE	CLUTCH
**Percent Hatched**	G = 11.999, p = 0.21	F_2,1_ = 0.558, p = 0.81
**Percent Survivorship**	**G = 36.61, p<0.0001**	**F_2,1_ = 4.848, p<0.05**
Mass	F_2,6_ = 0.510, p = 0.80	F_2,1_ = 2.007, p = 0.16
Curved Carapace Length	**F_2,6_ = 2.396, p<0.05**	F_2,1_ = 0.823, p = 0.37
**Righting Response**	**F_2,6_ = 2.969, p = 0.01**	**F_2,1_ = 8.761, p<0.01**
Crawl 1	F_2,6_ = 0.463, p = 0.83	**F_2,1_ = 4.360, p<0.05**
Crawl 2	F_2,6_ = 1.768, p = 0.13	F_2,1_ = 0.661, p = 0.52
**Crawl 3**	**F_2,6_ = 3.979, p<0.01**	F_2,1_ = 0.146, p = 0.86
**Change in Crawl Speed**	**F_2,6_ = 7.010, p = 0.01**	**F_2,1_ = 3.483, p<0.05**
**Overall Swim Activity**	**F_2,6_ = 8.022, p<0.0001**	*F_2,1_ = 4.686, p = 0.038*
%Time PowerStroke	F_2,6_ = 1.872, p = 0.11	F_2,1_ = 0.016, p = 0.90
%Time Rear Flipper Kick	F_2,6_ = 0.985, p = 0.45	F_2,1_ = 3.253, p = 0.080
%Time Mixed Rest	*F_2,6_ = 4.590, p = 0.001*	*F_2,1_ = 3.875, p = 0.057*
%Time Rest	*F_2,6_ = 3.106, p = 0.015*	F_2,1_ = 0.824, p = 0.37
Switch from PowerStroke	F_2,6_ = 0.210, p = 0.96	*F_2,1_ = 6.050, p = 0.019*
Avg. Stroke Rate Freq.	F_3,6_ = 0.887, p = 0.52	F_3,1_ = 0.190, p = 0.67
Approx. Distance Index	F_3,6_ = 1.262, p = 0.31	F_3,1_ = 0.073, p = 0.79
Change in Stroke Rate Freq.	mult.R^2^ = 0.090, p = 0.22	mult.R^2^ = 0.090, p = 0.22
Swim Speed Hour 1	F_2,6_ = 1.112, p = 0.37	F_2,1_ = 1.887, p = 0.178

Bold variables in the left-hand column indicate where there are figures presenting the result. Bold test statistics and p-values indicate a significant effect of either incubation temperature or clutch on the performance variable. Items in italics indicate a slight effect of either incubation temperature or clutch on the performance variable that did not meet the significance requirement after performing a Sidak-Bonferroni correction.

**Figure 6:**
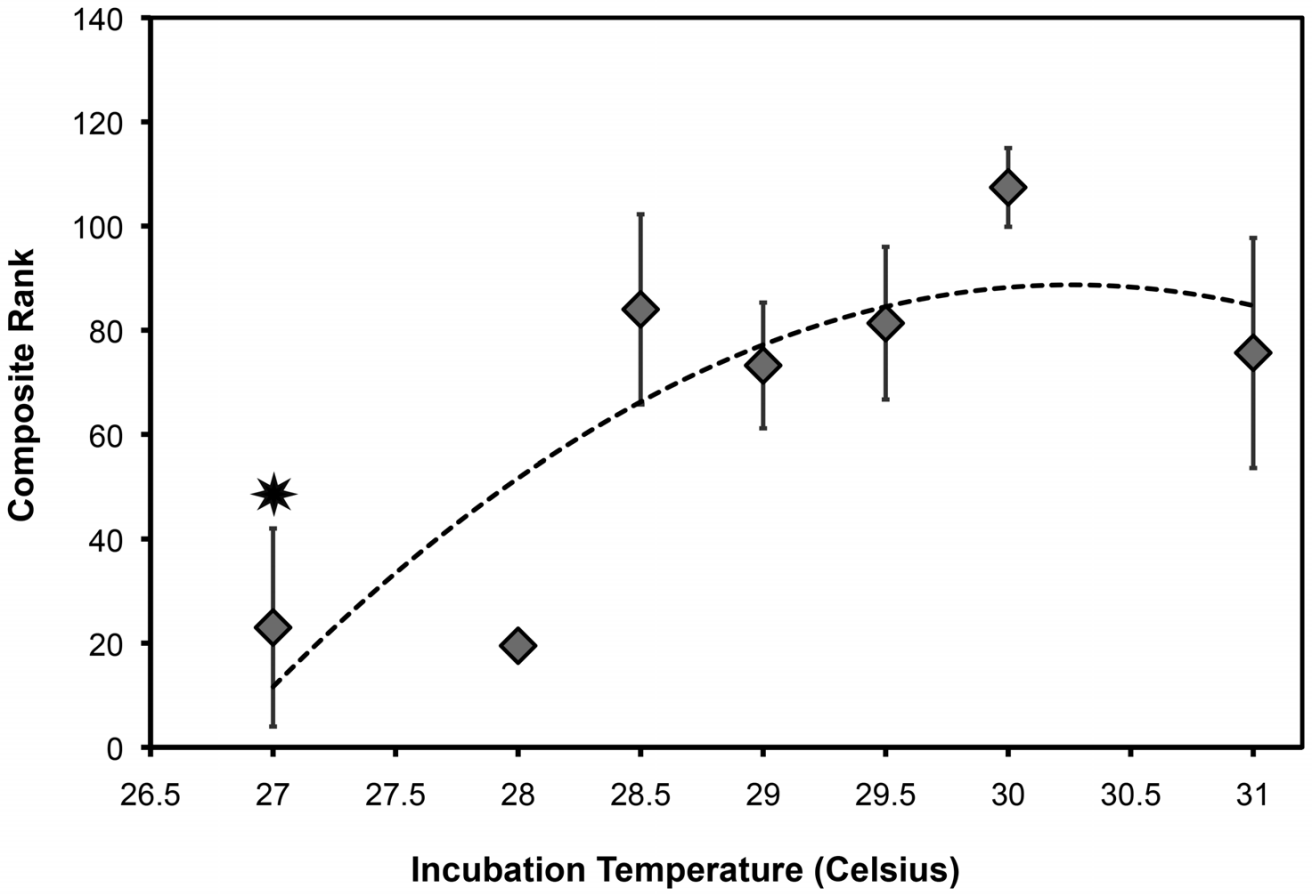
The composite rank for each incubation temperature ± S.E.M., fit with a quadratic curve through the means (y = −7.256x^2^+439.13x−6555.2; R^2^ = 0.70). Incubation temperature maintained a significant effect on the composite rank of hatchlings from 27°C (star). [Sample size: 27°C n = 4; 28°C n = 2; 28.5°C n = 7; 29°C n = 14; 29.5 n = 10; 30°C n = 6; 31 n = 4].

The composite rank value was derived by summing the ranks of each RR, C3 speed, change in crawl speed (C1-C3), overall swimming activity, and %MR value. These variables were considered independent measures of performance because there were no significant pair-wise relationships between each of these variables, with only a slight correlation between C3 speed and overall swim activity (R^2^ = 0.2). Hatchlings from 27°C retained the lowest rank, with the curve leveling off around 30°C (Fig. 6).

There was no discernible difference between AIC_c_ fit values or the nonlinear regression results for composite rank between a linear, quadratic, or 3^rd^ order polynomial function (see Table 2). The Akaike weight, or relative weight of evidence for each model, is highest with the quadratic fit (Table 2); however, this value remains close to the relative weight of the linear model. Despite these similarities between model fits, a quadratic function was chosen to fit the data based on the survivorship result of hatchlings incubated above 31°C. Hatchlings incubated at 32 and 32.5°C were too weak to survive the first few days of life, leading to the conclusion that if they had survived, they would have exhibited poor performance. Thus, a quadratic function was chosen for its potential predictive power to estimate composite rank on new data in the future (F_1,44_ = 4.671, p = 0.01, Fig. 6).

**Table 2:** Summary of the AIC_c_ values and fit of models applied to composite rank data.

MODELS OF FIT	AIC_c_ VALUE	AKAIKE WEIGHT
Linear	491.314	0.387
**Quadratic**	**491.144**	**0.421**
3^rd^ Order Polynomial	492.708	0.193

Although there is no discernable difference between AIC_c_ values, the quadratic function is 1.08 times more likely to be a better fit than the linear model, and 2.18 times more likely than the polynomial function.

In 2011, clutch of origin had a significant effect on righting response time (F_2,1_ = 8.761, p<0.01), C1 speed (F_2,1_ = 4.360, p<0.05) and change in crawl speed (F_2,1_ = 3.483, p<0.05). There were effects of clutch on overall swim activity (F_2,1_ = 4.686, p = 0.038) and first switch from PS (F_2,1_ = 6.050, p = 0.019). These results are summarized in Table 1.

### 2012: Incubation Temperature Effect on Performance Variables

Performance results from 2012 were kept separate from 2011 results for two reasons. First, there was an apparent temporal or year effect on the data that cannot be wholly assigned to a clutch difference; when data were normalized across clutches, significant differences remained between 2011 and 2012. Secondly in 2012, the overall temperature range was different from 2011: from 26.5–33°C. Furthermore, the actual temperature groupings and most frequently seen temperatures on shelves were different from 2011. Thus the results from 2011 and 2012 are not directly comparable.

The hatchlings that survived to be tested in 2012 were all incubated within the middle range of incubation: at 28, 29, 29.5, 30, and 30.5°C. At the lower temperature extreme, 26.5 and 27.5°C, sample sizes were too small to be included in the analyses (n = 1; n = 2, respectively). To repeat the Percent Hatched and Survivorship results described above, no hatchlings incubated at 32°C and above survived to be tested; this result was seen in both years for all clutches. Hatchlings from Clutch C incubated within this middle range of incubation temperatures showed no significant difference in performance at all performance tests (for righting response and crawl, p>0.3; for swim performance, p>0.03). These results support the performance results from 2011, where no significant differences in performance were seen in the middle range of incubation temperatures. A significant difference in performance was only observed at the cold extreme (27°C) as compared to the rest of the incubation temperatures. Due to the lack of significant differences in performance variables in 2012, no composite analysis was performed.

## Discussion

Using a controlled laboratory experiment manipulating incubation temperature, we observed an effect of incubation temperature on hatchling loggerhead performance, consistent with findings in previous hatchling sea turtle performance studies [5], [6], [7], [29], [39], [47], [58]. In general, turtles incubated at 27°C showed decreased locomotor ability; specifically they took longer to right themselves, crawled slower, slowed down while crawling, and swam for a shorter amount of time. Turtles incubated at 31°C took longer to right themselves but showed high locomotor ability in other tests. Turtles incubated at both 27°C and 31°C were also the smallest in curved carapace length. Hatchling turtles with decreased locomotor abilities have typically been interpreted to have a reduced chance of survival, and thus a lower overall fitness [20], [30].

### Hatch Success and Survivorship

For 2011 and 2012, both percent hatched and survivorship of hatchlings peaked around 29°C, in the middle of the incubation temperature range for loggerheads (Fig. 1). We saw survivorship decrease both towards the hot and cold ends of incubation temperature where sex ratio extremes would be seen; however, we did not anticipate that no hatchlings would survive at 32°C and 32.5°C (Fig. 1). Other studies have reported decreased hatch success at the hot and cold temperature extremes [30], [37], [38], [40], but other loggerhead incubation studies have seen relatively high hatch rates across a range of incubation temperatures (e.g., [36], [45], [58]). These studies only quantified hatch success; they did not measure survivorship of the hatchlings.

There is a possibility that experimental handling decreased survivorship of hatchlings in 2011 incubated at 32°C and 32.5°C. Eggs incubated at the warmest temperatures finished incubation before other temperatures, and when the first three animals had hatched, we did not allow them 24–36 hours to sit in their eggs before transporting them to the holding containers. Protocols were modified for subsequent hatchlings once mortality was observed. However, it remains possible that those first three hatchlings would have died based on the matching zero survivorship seen in 2012 for all hatchlings incubated at the same temperatures.

The overall percent hatched in 2011 at 62% and in 2012 at 33% were both lower than expected based on a previous study incubating loggerhead eggs from Florida, Georgia, and North Carolina, where percent hatched ranged from 72–96% [44]. It is possible that the egg transport from North Island, South Carolina to Morehead City, North Carolina affected development of the eggs, a theory supported due to the early-stage embryonic death seen for the majority of the unhatched eggs. However great care was taken during transport; once the eggs were removed from the beach, they were transported by boat back to land, where wave action could have disturbed the embryos, and they were also transferred between three vehicles. Previous studies have maintained a high hatch success following a lengthy transport (e.g., [36]: from Brazil to Canada, hatch success 93.4%), so another possibility is that a bacterial or fungal infection affected hatching success.

### Performance+Incubation Temperature

As predicted, decreased locomotor ability was seen for hatchlings incubated at the extremes of incubation temperature (Fig. 6). This was a uniform decrease at all performance measures for animals incubated at 27°C in 2011, indicating that suboptimal hatchlings are produced below a certain incubation temperature threshold. These results match controlled incubation studies conducted on green sea turtle hatchlings [4], [7] and many natural incubation studies [5], [6], [29], [39], [47], [58]. The results of our study and others support a classic reaction norm where mid-range incubation temperatures produce sea turtle hatchlings with optimal locomotor performance, whereas at the upper and lower extremes of viable incubation temperatures, hatchlings are produced displaying suboptimal locomotor performance. Poor performance from animals incubated at controlled low temperatures has also been seen for freshwater turtle hatchlings of several species [4], [13], [15]. For the Ouachita map turtle incubated at 25°C, a slower righting response was still observed after one year [15]. This reaction norm has significant implications for sea turtles, implying that greater survival is seen for hatchlings from incubation temperatures producing roughly even sex ratios of males and females.

At the warmer end of incubation temperatures tested in 2011, hatchlings produced at 31°C were seen to have a decreased ability to right themselves (Fig. 2). However, they maintained locomotor ability while crawling and swimming (Figs. 3–5). This suggests that the upper threshold for suboptimal loggerhead hatchlings begins around 31°C; however, the lack of survivorship at 32°C and above for both years prevented us from testing performance of these animals. This has been confirmed in the field for South Pacific loggerhead hatchlings, who performed the worst in terms of righting time and crawl speed when incubated around 32°C and 32.5°C [58]. For freshwater turtles, the Mary River turtle (*Elusor macrurus*) showed decreased performance ability when incubated at a controlled elevated temperature of 32°C; specifically these animals took 30 times longer to right themselves, swam for less amount of time, and exhibited less stroke force output [40]. No controlled studies on loggerheads have tested hatchlings produced at 32°C, but based on field studies of loggerhead hatchling performance and our result of reduced survivorship at warmer temperatures two years in a row, we would expect to see similar results [47], [58].

The righting response test has recently been used on sea turtle hatchlings [6], [27], [38], [39], [47], [52], [58], and it has been frequently used on freshwater turtle hatchlings to estimate fitness (e.g. [12], [15], [28], [40], [53]). Results from righting response studies have not always correlated with incubation temperature for all freshwater turtle species, for example in the snapping turtle (*Chelydra serpentina*) [53]. The righting response is most likely not an exact fitness estimate; however, in our study and two in-field studies, this simple and fast test showed a significant effect of incubation temperature on loggerhead hatchlings, and it is easily replicable both in laboratories and in the field [47], [58]. Furthermore, features on a beach can cause hatchlings to find themselves upside down on their carapaces, so the ability to flip back over could be critical to surviving the crawl to the ocean [27]. We encourage the continued use of the righting response test on sea turtle hatchlings to facilitate research on the effects of incubation temperature in more natural settings.

The Composite Rank measure is a novel way to estimate overall hatchling fitness based on the performance measures tested in this study. This type of analysis can be categorized as a “Performance Index”, and provides a uniform way to compare hatchlings using multiple correlates of survival [24]. Although different curves fit equally well based on AIC_c_ values, the quadratic function has the highest Akaike weight and best accounts for the other performance results as well as our ecological understanding of incubation temperature effects on hatchling performance. In other words, the shape of the curve for a linear or 3^rd^ order polynomial function does not reflect the effects of incubation temperature on performance observed for this species, whereas a quadratic curve agrees with trends observed in each separate performance variable.

### Implications

Based on the hatch success, survivorship, and performance results in 2011 and 2012, the optimal range of hatchling loggerhead production appears to be at incubation temperatures from approximately 28.5–31°C. The decreased performance ability seen in hatchlings incubated at 27°C in 2011 may indicate why loggerheads do not nest frequently north of North Carolina. It has been suggested for snapping turtles (*Chelydra serpentina*) that incubation conditions limit the species range, and the poor condition of loggerhead hatchlings born at these low temperatures supports this theory [1].

Loggerhead nesting on the Atlantic coast of the southeastern United States occurs on beaches from Florida to North Carolina, although the majority of nesting occurs in Florida [57]. Interestingly, incubation temperatures for most of the nesting season in Florida appear to be higher than 31°C [23], [33]. North of Florida, nest temperatures may reach 32°C and above during the summer [32]. At temperatures above approximately 32°C, an increase in morphological abnormalities and decrease in hatching success are seen [42], [43]. Furthermore, when sand temperatures on the beaches are around 32°C, the actual nest temperature may be warmer due to metabolic warming. Metabolic warming can cause hatchling death when the metabolism of hatchlings generates enough heat in the nest to raise nest temperature to lethal levels [37]. This production of weakened hatchlings and even hatchling death has been reported globally, in Australia [8], [34], Japan [37], Indonesia [38], North Carolina [11], and Mexico [50].

Due to the conditions described above, it is feasible that many beaches along the southeastern United States are producing millions of loggerhead hatchlings that perform sub-optimally. With the onset of climate change and warming sand temperatures along the Atlantic coast, this could result in an even greater production of suboptimal hatchlings, and an increase in nest mortalities from hot incubation temperatures [26]. These effects will not facilitate the recovery of the Northwestern Atlantic loggerhead and could already be affecting population dynamics.

To test this hypothesis, we recommend that similar performance testing should be done on hatchlings produced on Florida nesting beaches to test if there is geographic variation in performance ability. It is important to quantify hatching success and survivorship in the field, because these numbers could differ from our laboratory incubation results. It is also critical to incorporate more clutches in future experimental designs, to better evaluate any maternal effects on hatching success, survivorship, and performance, which have been seen in other studies [6]. The three clutches tested in this study were from South Carolina nesting females, and it is possible that there is heritable variation resulting in a lowering of the upper incubation temperature limit for these eggs. In the literature, incubation temperatures higher than ∼33°C are listed as lethal for loggerhead eggs [42], but the results of our study suggest that incubation temperatures of 32°C or above may drastically decrease hatch success and survivorship for South Carolina loggerheads. It would be interesting to see if there is a difference in this upper limit between Florida-originated hatchlings and the Northern subpopulation.

### Beyond Controlled Incubation

From a conservation standpoint, our use of controlled incubation temperatures makes it challenging to extend these results to hatchlings produced in a natural incubation environment with temperatures that commonly fluctuates (e.g., [23]). However, this experiment is an important first step in better understanding the basic effect incubation temperature has on loggerhead hatchling performance. In our study we sought to address this question by controlling as many incubation variables as possible, except for incubation temperature. Other studies have demonstrated the same effects of incubation temperature on phenotype and performance both in the laboratory and in the field [41], [51]. Furthermore, our results support locomotor performance results seen for South Pacific loggerhead hatchlings produced in a natural incubation environment [47], [58]. It is important to build off of these laboratory results for Atlantic loggerheads and subsequently investigate hatchlings' performance abilities in the field.

For green sea turtle hatchlings, studies either based in the field or that included fluctuating incubation temperatures in the laboratory have reported an increase in variation of performance ability [5], [29]. We would predict that fluctuating temperatures could increase loggerhead performance ability, but like the green sea turtle hatchlings, this scenario would also introduce more variation. In our study, hatchlings from the single incubator that fluctuated ∼2°C in 2011 showed the most variation for many of the performance measures (data not shown). This could be explained ecologically, if fluctuating incubation temperatures in the wild produce hatchlings with a wide range of performance abilities. It may decrease the survival chances for some, but increase the fitness of others, thereby potentially increasing the mother's lifetime fitness. Furthermore, fluctuating incubation temperatures may help offset the potentially damaging effects of constant temperatures at or above 32°C.

Future studies testing Atlantic loggerhead hatchling performance could start by continuing laboratory incubations to control certain variables, but build in fluctuating incubation temperatures; then based on these results, hatchlings should be tested from natural nests. The results of these studies, building off of the results from our controlled experiments, would provide a more complete picture of the natural effects of incubation temperature on the early life history of the loggerhead sea turtle. In the face of climate change and diminishing sea turtle populations, this information could be critical to inform management of nesting beaches in coming years.
